# Self-thermophoresis of laser-heated spherical Janus particles

**DOI:** 10.1140/epje/s10189-021-00128-4

**Published:** 2021-11-17

**Authors:** E. J. Avital, T. Miloh

**Affiliations:** 1 School of Engineering and Materials Science, Queen Mary University of London, London, E1 4NS UK; 2 School of Mechanical Engineering, Tel Aviv University, Tel-Aviv, 69978 Israel

## Abstract

**Abstract:**

An analytic framework is presented for calculating the self-induced thermophoretic velocity of a laser-heated Janus metamaterial micro-particle, consisting of two conducting hemispheres of different thermal and electric conductivities. The spherical Janus is embedded in a quiescent fluid of infinite expanse and is exposed to a continuous light irradiation by a defocused laser beam. The analysis is carried under the electrostatic (Rayleigh) approximation (radius small compared to wavelength). The linear scheme for evaluating the temperature field in the three phases is based on employing a Fourier–Legendre approach, which renders rather simple semi-analytic expressions in terms of the relevant physical parameters of the titled symmetry-breaking problem. In addition to an explicit solution for the self-thermophoretic mobility of the heated Janus, we also provide analytic expressions for the slip-induced Joule heating streamlines and vorticity field in the surrounding fluid, for a non-uniform (surface dependent) Soret coefficient. For a ‘symmetric’ (homogeneous) spherical particle, the surface temperature gradient vanishes and thus there is no self-induced thermophoretic velocity field. The ‘inner’ temperature field in this case reduces to the well-known solution for a laser-heated spherical conducting colloid. In the case of a constant Soret phoretic mobility, the analysis is compared against numerical simulations, based on a tailored collocation method for some selected values of the physical parameters. Also presented are some typical temperature field contours and heat flux vectors prevailing in the two-phase Janus as well as light-induced velocity and vorticity fields in the ambient solute and a new practical estimate for the self-propelling velocity.

**Graphic abstract:**

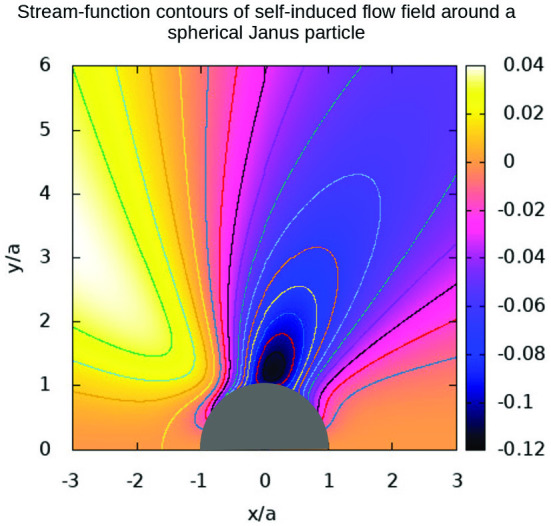

## Introduction

The subject of self-propelled autonomous micro-/nano-motors and micro-swimmers has recently gained much attention due to its vast applications in various fields such as nanotechnology, biology and medicine [1]. Self-propulsion mechanism in micro-systems is generally associated with the conversion of some local chemical (catalytic) energy source [2] or with different means of external incitements, such as imposed electric and magnetic fields, ambient concentration and temperature gradients, ultrasonic and acoustic waves as well as light-induced Joule heating [3, 4]. Much attention has been devoted to laser-heated or light–induced thermophoretic transport of catalytic [5–7] and non-catalytic micro-motors (see recent reviews [8–11]). Achieving a finite thermophoretic velocity of a light-activated homogeneous symmetric colloid (e.g. sphere) is possible by introducing some sort of physical/chemical ‘symmetry-breaking’ effect. For active-catalytic particles, it may arise from imposing a non-uniform chemical reaction on its surface [2] or from a genuine metamaterial asymmetry such as a ‘two-phase’ hot Janus particle (JP), e.g. [12–14].

Thermophoresis is generally a process where a particle is propelled by an imposed temperature gradient in the solute [15–21] or by a Soret-type force due to temperature variations (non-uniformity) over its surface. Joule heating enhancement can be also achieved under localized thermoplasmonic resonance conditions [4, 22–24]. The induced temperature variation over the colloid surface, results in a thermoosmotic velocity slippage, where the tangential velocity is proportional to the surface temperature gradient (Soret effect) and to a prescribed surface-dependent mobility coefficient. Integrating this velocity over the wetted smooth particle surface, leads to a finite thermophoretic velocity [20, 25–27].

For practical reasons, much attention is directed towards ‘self-thermophoresis’, where the temperature variation along the particle is not caused by imposing an external temperature gradient, but rather due to some material or physio-chemical asymmetry (symmetry-breaking) within the particle itself or along its surface in the case of catalytic colloids [7]. One way to achieve this is to use photoactive (light-activated) spherical JP’s, consisting of two hemispheres of different thermal and electric conductivities, which is the focus of this study. The term of ‘self-thermophoresis’, was first coined in [12], which examined a spherical JP by half-coating its surface with a thin layer of metal (Ag). The concept of self-thermophoresis is similar in many ways to ‘self-dielectrophoresis’ [28] and ‘self-diffusiophoresis’ [29], whereby a non-uniform electric field is generated by a material Janus asymmetry exposed to an ambient uniform electric field, resulting in a finite colloid mobility.

The present analysis is related and complements the recent works of [7, 15] on thermotaxis of a light-activated JP by considering a non-catalytic Janus and in trying to analytically determine its thermophoretic mobility and thermoosmotic flow field in terms of the corresponding physical parameters using first principles. For comparison, the temperature field on the JP surface in [14] is assumed to be induced by a nearby heat source (modelled by a highly conducting gold nanoparticle of 250 nm). The surface temperature gradient is then evaluated numerically (Comsol) assuming a piecewise constant Soret slip and the resulting thermophoretic velocities (linear and angular) and JP’s trajectories are found. However, we consider here the fundamental problem of a non-catalytic JP with a surface-dependent (non-uniform) mobility, where symmetry-breaking results from disparate Joule heating effects or temperature distributions existing within the two hemispheres (of different thermal and electric conductivities). We thus consider JP ‘point’ heating by a defocused laser, in accordance with Rayleigh’s assumption [4] and are able to obtain explicit expressions for the temperature field in the three phases as well as for the velocity field around the JP, determining its thermophoretic mobility. Note that due to the axially symmetric temperature field induced in the JP, there is no dependence on laser directionality (no JP rotation). It is also worth mentioning that in the current analysis the asymmetric temperature distribution induced on the JP surface, does not depend on any *external* forcing and is instead generated due to internal Joule heating mechanisms within each hemisphere. Different temperature fields are thus induced in the (two) solid and fluid phases, depending on the corresponding values of the thermal and electric conductivity of each phase as well as on the amount of light irradiation (laser power). We are thus able to obtain a rather simple and functional new analytic solution for the fundamental problem involving self-thermophoretic mobility of a photoactive JP in terms of the relevant physical parameters, using the common thermal boundary conditions and Soret-type slip, enforced on the various JP interfaces.

A somewhat similar approach for a coated JP has been employed in [13], by arguing that the temperature variations inside the particle are *uniform* and by utilizing an ad hoc jump function across the particle’s inner interface. An approximate solution for the temperature field was then obtained for two limiting cases, namely for ‘thin’ and ‘thick’ coatings. A Fourier–Legendre (FL) series was also used to model the temperature and induced velocity fields in the fluid (assuming a creeping flow). The JP mobility was then found to depend only on the bipolar term of the external temperature series. In the process of obtaining the approximate solution, it was assumed that the thermal conductivity of the JP and the ambient solute are the *same* and no dependence on the electrical conductivity of the two phases was taken into account. However, our solution is *exact* in the sense that it analytically determines the non-uniform temperature field within the JP and it also accounts for the disparity between the thermal and electric conductivities existing in the three-phase problem.

The self-thermophoretic problem of a half-coated JP with a layer of a different material at the low salinity limit has been also considered in [30] , by employing again the inner ‘uniform’ temperature assumption of [13]. Solutions for the temperature fields were derived analytically and computationally using a finite element method. The case of a photoactive spheroidal JP, assuming a large contrast (i.e. ideally dielectric and perfectly conducting halves) has been analytically discussed in [14]. Both inner and outer temperature fields were explicitly obtained under these restrictive conditions and the JP’s mobility, depending on its eccentricity was also obtained. The present work can be considered as a generalization of [14] by providing an analytic solution for the thermotaxis problem of a spherical JP by considering a full three-phase configuration with different physical/chemical parameters and by applying the proper thermal boundary conditions on the relevant JP interfaces. It is interesting to note that the self-induced thermophoretic mobility of orthotropic (e.g. sphere, spheroid, ellipsoid*) homogeneous* light-activated particles is always null! Nevertheless, except for a perfectly symmetric sphere, laser-heated spheroidal and ellipsoidal shapes generally induce *finite* dipole-type (symmetric) thermoosmotic velocity and vorticity fields in the surrounding electrolyte [31].

An experimental study of self-propelled JP’s was pursued by [32], by using a homogeneous spherical particle having a cube of another material attached to it. Trajectory loops were created using a diverging laser beam. An interesting Janus-like configuration was also proposed [33], having pairs of spherical particles, where each pair consisted of two particles of different materials and providing estimates for the thermophoretic forces and velocities. This brings us to the idea of designing special micro-motors based on the JP concept (e.g. [8]), where particles are integrated with shells of different materials or manufactured layer by layer to achieve the desired JP asymmetry. Partly coated particles were also manufactured by [34], who experimentally used laser irradiation to demonstrate control on the collective behaviour of such particles.

Despite the current growing research activity in self-thermophoresis of JP’s, there are still some open research questions that affect our understanding and ability to optimally manufacture autonomous micro-motors based on the JP concept. Commonly, a uniform temperature distribution inside the different zones of the JP is assumed along with an ad hoc jump function to account for the different zones’ temperatures. Such an approximate solution clearly does not fulfil the physical boundary conditions ensuring continuity of both temperature and heat flux across the different JP interfaces. In addition, these approximations do not specifically depend on the various thermal and electric parameters of the corresponding three distinct phases. Thus, there is a pressing need for developing a more general analytic solution that can affectedly account for the large disparity between the different physical coefficients of the three-phase media, while preserving the proper physical (thermal) interfacial boundary conditions.

**Fig. 1:**
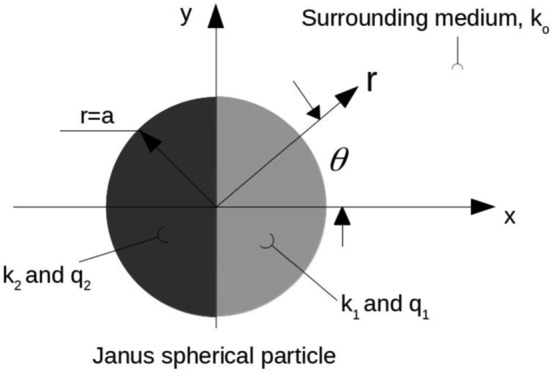
Schematic description of the Janus particle (JP) problem, where *r* is the spherical radius, the JP radius is *a* and $\theta$ is the spherical angle

As demonstrated by [35] for laser-heated homogeneous spheroidal particles, the difference between the particle’s thermal conductivity and the surrounding conductivity of the liquid, can much affect the patterns of the surface heat flux and the induced thermoosmotic flow. Therefore, one expects that a sharp contrast between the thermal conductivities of the JP hemispheres should have a considerable effect on both the ‘inner’ and ‘outer’ temperature distributions and as a result also on the induced self-thermophoretic velocity field. For this goal, we have looked at a single freely suspended spherical JP composed of two halves with different material properties as illustrated in Fig. 1. The Fourier–Legendre (FL) series technique, that was proven useful in previous studies, was used here as well to determine the temperature fields in each hemisphere The linearized ‘symmetry-breaking’ physical model is first discussed in Sect. 2 by specifying the different governing equations in each phase and the thermal boundary conditions applied on the various interfaces. Details of using the FL methodology are next outlined in Sect. 3 and the appendices. Some discussions of the analytic results and numerical simulations thus found are presented in Sects. 4 and 5, followed by a conclusion section.

## The linearized ‘symmetry-breaking’ physical model

We consider a spherical JP of radius *a* consisting of two hemispheres of different thermal conductivities ($\hbox {k}_{{1}}$, $\hbox {k}_{{2}})$ and corresponding heat sources ($\hbox {q}_{{1}}$, $\hbox {q}_{{2}})$, as illustrated in Fig. 1. The particle is freely suspended in a dielectric medium with a thermal conductivity $\hbox {k}_{\mathrm {O}}$ with no external heat source. The axis of symmetry x is taken to be normal to the interior JP interface . Using spherical (axisymmetric) coordinates $\left( r,\theta \right) ,x=r\, cos\theta$, the interior circular interface between the two hemispheres is given by $\mu =cos\theta =0$ and $0\leqslant r \leqslant a$.

When the JP is irradiated by a defocused laser at a wavelength much larger than the particle’s radius (Rayleigh’ assumption), the temperature field in each JP phase can be estimated using the following linear Poisson equation [13, 22, 31];

1 $$\begin{aligned} \nabla ^{2}T_{i}=-q_{i}, \end{aligned}$$

where  and $i=1, 2$. Here $\sigma _{i}$ denotes the electrical conductivity of each hemisphere,  is the volume of the spherical particle, and $| \hbox {E}_{{0}} |$ is the amplitude of the electric field (laser) which is assumed to be uniform inside the micron-sized JP.

The thermal conductivities and internal heat sources of the JP hemispheres are generally different, hence ${T}_{{1}}$ differs from ${T}_{{2}}$. Both temperature fields must fulfil continuity in temperature and heat flux across the interior JP interface leading to the following boundary conditions at $\mu =0,~ 0\leqslant r\leqslant a$:

2 $$\begin{aligned}&T_{1}=T_{2}, \end{aligned}$$

3 $$\begin{aligned}&k_{1}\frac{\partial T_{1}}{\partial \mu }=k_{2}\frac{\partial T_{2}}{\partial \mu }. \end{aligned}$$

Next, assuming that the outer fluid domain is practically non-conducting, implies that the outer temperature ${T}_{\mathrm {O}}$ is harmonic, i.e. $\nabla ^{2}T_{\mathrm {O}}=0$. Yet, on the JP surface $r=a$, one also needs to satisfy continuity in the temperature and heat flux normal to the surface on each hemisphere [35];

4 $$\begin{aligned}&T_{\mathrm {O}}=T_{i}, \end{aligned}$$

5 $$\begin{aligned}&k_{\mathrm {O}}\frac{\partial T_{\mathrm {O}}}{\partial r}=k_{i}\frac{\partial T_{i}}{\partial r}, \end{aligned}$$

where $$i=1,2$$.

The resulting non-uniform temperature field along the JP surface, will generate an induced thermophoretic velocity field around the particle. The induced slip velocity $\overrightarrow{v_{S}}$ over the particle’s surface, following the boundary layer approximation of Anderson [18], is related to the temperature gradient surface gradient $\left( \nabla _{S} \right)$ by the Soret-type slip condition [12, 15, 17, 18, 29, 31];

6 $$\begin{aligned} \overrightarrow{v_{S}}=-D_{\mathrm {T}}\nabla _{S}T_{\mathrm {O}}. \end{aligned}$$

The surface-dependent parameter $D_{\mathrm {T}}$, represents the thermophoretic mobility (Soret coefficient). It can depend on the temperature and composition of the JP surface [25]. Jiang et al. [12] argued for approximating it as a constant for JP due to a small temperature variation over the JP surface. In Sects. 3 and 4, we follow this suggestion and approximate $\hbox {D}_{\mathrm {T}}$ as constant. Nevertheless, in ‘Appendix B’ a full solution is given for the velocity field for a variable $\hbox {D}_{\mathrm {T}}$ depending on the polar angle. Furthermore in Sect. 5, when a comparison is made to the literature available experimental results of a JP created by thin coating on one of the hemisphere, it is shown that $\hbox {D}_{\mathrm {T}}$ varies by up to 20% (around the average) for the extreme case of a JP composed by gold and polystyrene. This supports the approximation of $\hbox {D}_{\mathrm {T}}$ as constant for the JP of Beryllium–Copper and polystyrene in Sect. 4. The induced thermoosmotic velocity field in the surrounding liquid is taken as Stokes type due to the low Reynolds number caused by the small mobility and dimension of the JP.

## Solution methodology

Both the temperature and velocity fields are axisymmetric, because $k_{\mathrm {i}}$, $q_{\mathrm {i}}$ and $k_{\mathrm {O}}$ are taken as constants. The temperature distribution inside each JP hemisphere is expressed in terms of a FL series by distinguishing between odd and even terms as follows, where $R=r/\hbox {a}$;

7 $$\begin{aligned} T_{1}\left( R,\mu \right)= & {} {\overline{T}}_{1}\left( R,\mu \right) +\sum \limits _{n=0}^\infty A_{2n} R^{2n}P_{2n}\left( \mu \right) \nonumber \\&+\sum \limits _{n=0}^\infty A_{2n+1} R^{2n+1}P_{2n+1}\left( \mu \right) , \end{aligned}$$

8 $$\begin{aligned} T_{2}\left( R,\mu \right)= & {} {\overline{T}}_{2}\left( R,\mu \right) +\sum \limits _{n=0}^\infty A_{2n} R^{2n}P_{2n}\left( \mu \right) \nonumber \\&+\frac{k_{1}}{k_{2}}\sum \limits _{n=0}^\infty A_{2n+1} R^{2n+1}P_{2n+1}\left( \mu \right) , \end{aligned}$$

where

9 $$\begin{aligned}&{\overline{T}}_{1}\left( R,\mu \right) =-\frac{a^{2}}{6}\left[ q_{1}R^{2}+D+\left( q_{1}-q_{2} \right) R^{2}P_{2}\left( \mu \right) \right] ,\nonumber \\ \end{aligned}$$

10 $$\begin{aligned}&{\overline{T}}_{2}\left( R,\mu \right) =-\frac{a^{2}}{6}\left[ q_{2}R^{2}+D+\left( q_{2}-q_{1} \right) R^{2}P_{2}\left( \mu \right) \right] ,\nonumber \\ \end{aligned}$$

11 $$\begin{aligned}&D=-q_{1}\left( \frac{1}{2}+\frac{k_{1}}{k_{O}} \right) -q_{2}\left( \frac{1}{2}+\frac{k_{2}}{k_{O}} \right) , \end{aligned}$$

and $P_{n}\left( \mu \right)$ are the Legendre polynomials. The expressions in Eqs. (7)–(11) fulfil the governing equation (1) for the temperature field inside the particle. Also note that since $P_{2n+1}\left( \mu =0 \right) =0$, $P_{2}\left( \mu =0 \right) ={-1}/2$ and the way the series was split between the odd and even modes, Eqs. (7) and (8) automatically fulfil the JP interface thermal boundary conditions, as expressed in Eqs. (2) and (3).

The external harmonic temperature field ${T}_{\mathrm {O}}$ can be expressed accordingly as;

12 $$\begin{aligned} T_{\mathrm {O}}\left( R,\mu \right)= & {} \sum \limits _{n=0}^\infty C_{2n} R^{-\left( 2n+1 \right) }P_{2n}\left( \mu \right) \nonumber \\&+\sum \limits _{n=0}^\infty C_{2n+1} R^{-2\left( n+1 \right) }P_{2n}\left( \mu \right) . \end{aligned}$$

The unknown series coefficients ${A}_{{2n}}$, ${A}_{{2n+1}}$, ${C}_{{2n}}$ and ${C}_{{2n+1}}$ are calculated using the collocation method [36]. The JP surface is divided into N elements and the boundary conditions of Eqs. (4) and (5) requiring continuity in both temperature and heat flux normal to the surface to be matched at the centre point of each element, yielding a matrix equation of the same order. The matrix equation can be symbolically written as $M\cdot s=b$, where the vector *s* contains the coefficients ${A}_{{2n}}$, ${A}_{{2n+1}}$, ${C}_{{2n}}$ and ${C}_{{2n+1.}}$ The matrix equation can be solved by the well-known LU decomposition procedure [37].

A similar procedure but in the spectral space is outlined in ‘Appendix A’, with three interesting results that are repeated here. First is an analytic expression for $\hbox {C}_0$, as expressed in Eq. (A1). Second, an explicit closed-form solution for the temperature fields in the case $k_{1}=k_{2}$, but still with $q_{1}\ne q_{2}$, as expressed in Eqs. (A13) and (A14). Third is an explicit simple two-term (truncated) approximation for the ‘mobility’ coefficient ${C}_{{1}}$ as expressed in Eq. (A16). The approximate solution thus found, yields about 10% difference between the ‘precise’ ${C}_{{1}}$ computed by the collocation method. This is of particular importance, as the coefficient ${C}_{{1}}$ controls the self-thermophoretic velocity of the laser-heated JP as seen later in Eq. (13). Thus, one can easily estimate the magnitude and direction of the self-induced mobility of the hot JP in terms of the corresponding thermal and electrical parameters of the titled problem.

The thermoosmotic flow field induced around the JP is affected by the Soret-type slip velocity of Eq. (6). The asymmetry between negative and positive *x* directions, as illustrated in Fig. 1, will generate a self-propelling velocity $U_{\mathrm{{P}}}$ in the *x* direction. The mobility $U_P$, can then be calculated by averaging the component of $\overrightarrow{v_{S}}$ in the x direction over the JP. Substituting $\hbox {T}_{\mathrm {O}}$ given in Eq. (12) into Eq. (6) and averaging $\overrightarrow{v_{S}}$ over the JP surface, leads to;

13 $$\begin{aligned} U_{\mathrm{{P}}}=-\frac{2D_{\mathrm{{T}}}}{3a}C_{1}. \end{aligned}$$

Eq. (13) shows that the JP velocity depends only on the first odd term of the series solution of ${T}_{\mathrm {O}}$. It extends Bickel et al.’s [13] result that showed that the mobility of a partly coated JP depends only on the dipolar term of the outer temperature field.

Once the slip velocity $\overrightarrow{v_{S}}$ is known, the induced velocity field can be calculated assuming a Stokes-type flow due to the particle’s low Reynolds number [15]. Using Eq. (12) and accounting for the particle’s self-propelling velocity $U_{P}\hat{x}$ to derive the axisymmetric velocity field in a moving coordinate frame attached to the JP, one gets the following explicit expression for the 3D Stokes stream function $\psi$;

14 $$\begin{aligned} \psi \left( r,\theta \right)= & {} \frac{D_{\mathrm{{T}}}a}{2}{\mathrm{{sin}}}^{2}\theta \left\{ \frac{2}{3}C_{1}\left( \frac{a}{r} \right) \right. \nonumber \\&\left. -\sum \limits _{n=2}^\infty {C_{n}\left( \frac{a}{r} \right) ^{n-2}\left[ 1-\left( \frac{a}{r} \right) ^{2} \right] \frac{{\mathrm{{d}}P}_{n}\left( \mu \right) }{\mathrm{{d}}\mu }} \right\} ,\nonumber \\ \end{aligned}$$

and the corresponding expressions for the radial $v_{r}$ and tangential $v_{\theta }$ velocity components;

15 $$\begin{aligned} v_{r}\left( r,\theta \right)= & {} \frac{2D_{\mathrm{{T}}}a^{2}}{3r^{3}}C_{1}\mathrm{{cos}}\theta \nonumber \\&+\frac{D_{\mathrm{{T}}}a}{2r^{2}}\sum \limits _{n=2}^\infty n\left( n+1 \right) C_{n}\left( \frac{a}{r} \right) ^{n-2}\nonumber \\&\left[ 1-\left( \frac{a}{r} \right) ^{2} \right] P_{n}\left( \mu \right) , \end{aligned}$$

16 $$\begin{aligned} v_{\theta }\left( r,\theta \right)= & {} \frac{D_{\mathrm{{T}}}a}{2r^{2}}\mathrm{{sin}}\theta \left\{ \frac{2}{3}C_{1}\left( \frac{a}{r} \right) -\sum \limits _{n=2}^\infty C_{n}\left( \frac{a}{r} \right) ^{n-2}\right. \nonumber \\&\left. \left[ n-2-n\left( \frac{a}{r} \right) ^{2} \right] \frac{{\mathrm{{d}}P}_{n}\left( \mu \right) }{\mathrm{{d}}\mu } \right\} . \end{aligned}$$

Finally, taking in Eqs. (15) and (16) $r={a}$, leads to $v_{r}\left( r=a,\theta \right) =-U_{\mathrm{{P}}}\mathrm{{cos}}\theta$ and $v_{\theta }\left( r=a,\theta \right) =U_{\mathrm{{P}}}\mathrm{{sin}}\theta +\overrightarrow{v_{S}}\cdot \hat{\theta }$ as expected, in accordance with Eq. (13).

## Results and discussion for the developed JP model of two hemispheres

The general temperature solution procedure of the collocation method was coded and verified against the explicit solution given in ‘Appendix A’ for the special case of ${k}_{\mathrm {O}} < {k}_{{1}}= {k}_{{2}}$ and $q_{1}\ne q_{2}$. Further verification was carried out for the cases of ${k}_{{1}} = 8$ W/(m K) or 0.04 W/(m K), with, ${k}_{{2}} = {k}_{{1}}$ and $q_{1}\ne q_{2}$ against a finite-difference solution of the heat equation Eq.(1) using a method as described in [35], achieving an excellent agreement.

**Fig. 2:**
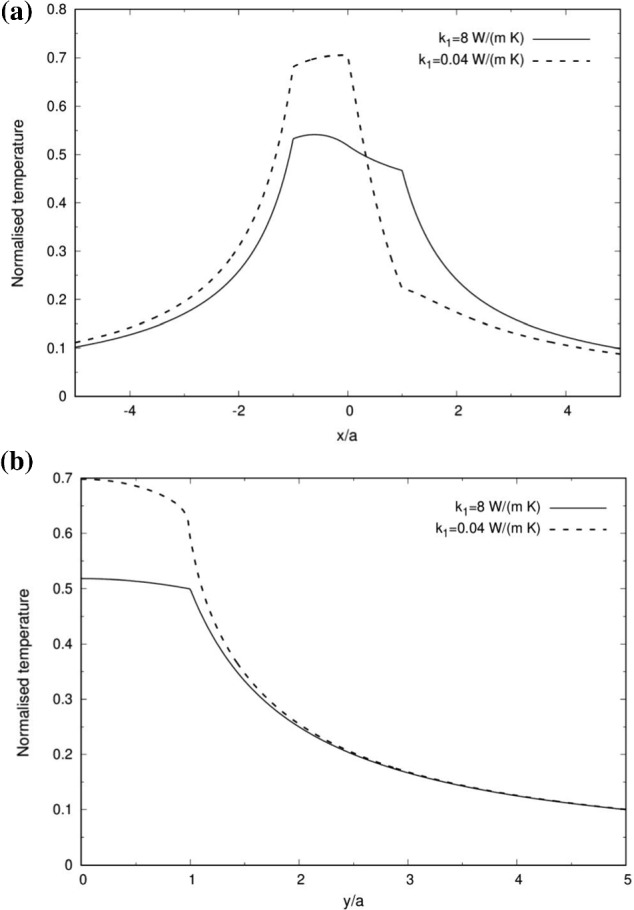
The variation of the temperature normalized by $$T_{ref}=q_{2}a^{2}k_{2}/\left( 3k_{o} \right) $$ along the particle’s (**a**) axis of symmetry and (**b**) the boundary axis between the two Janus particle’s halves, where $${q}_{{1}}= 0$$, $${k}_{\mathrm {O}}= 0.6$$ W/(m K) as of water and $${k}_{{2}} = $$ 8 W/(m K) as of Beryllium copper

**Fig. 3:**
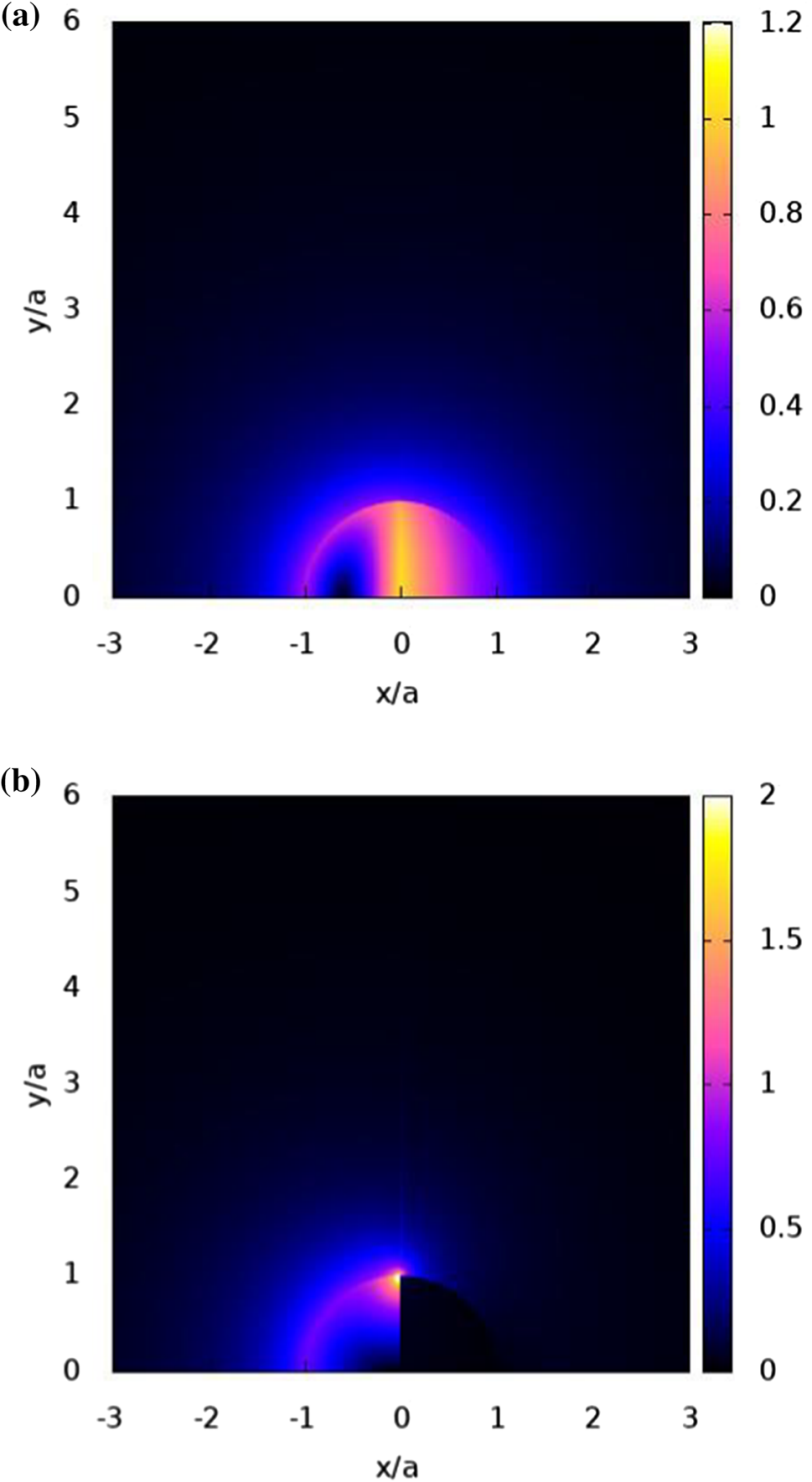
Contours of the heat flux modulus normalized by $q_{2}{ak}_{2}/3$ and which are plotted for the Janus particles of Fig. 2

**Fig. 4:**
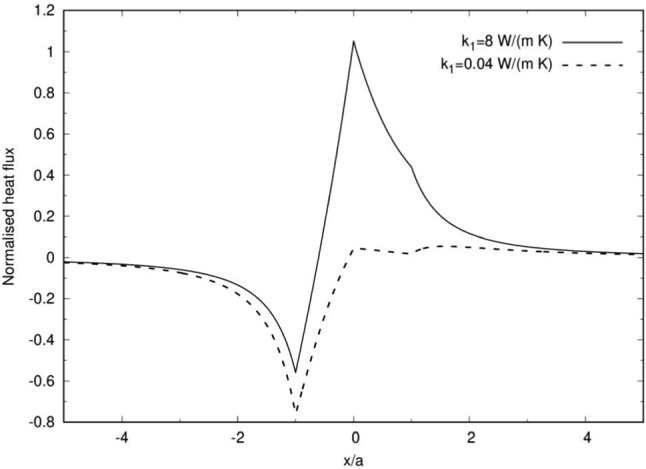
The normalized heat flux that is plotted along the particle’s axis of symmetry *x*. The rest of the conditions are as in Fig. 3

**Fig. 5:**
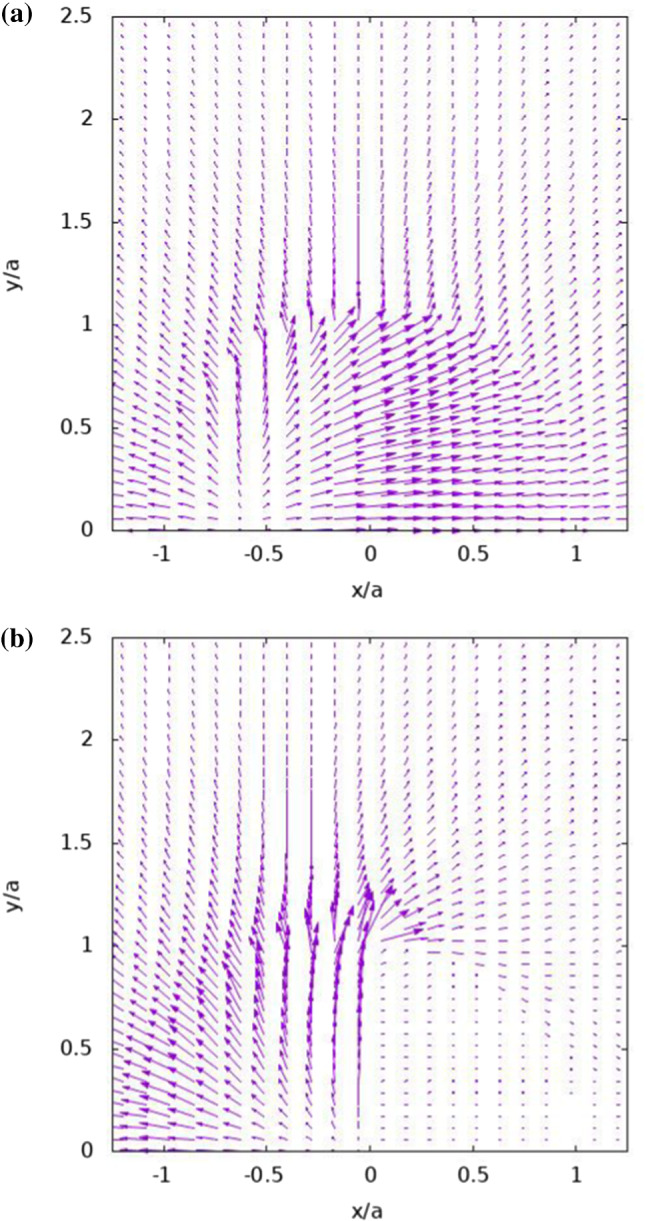
Heat flux vectors that are plotted for the cases of Fig. 3, where the vector modulus of each field was scaled for optimal presentation and each vector is located at its tail

**Fig. 6:**
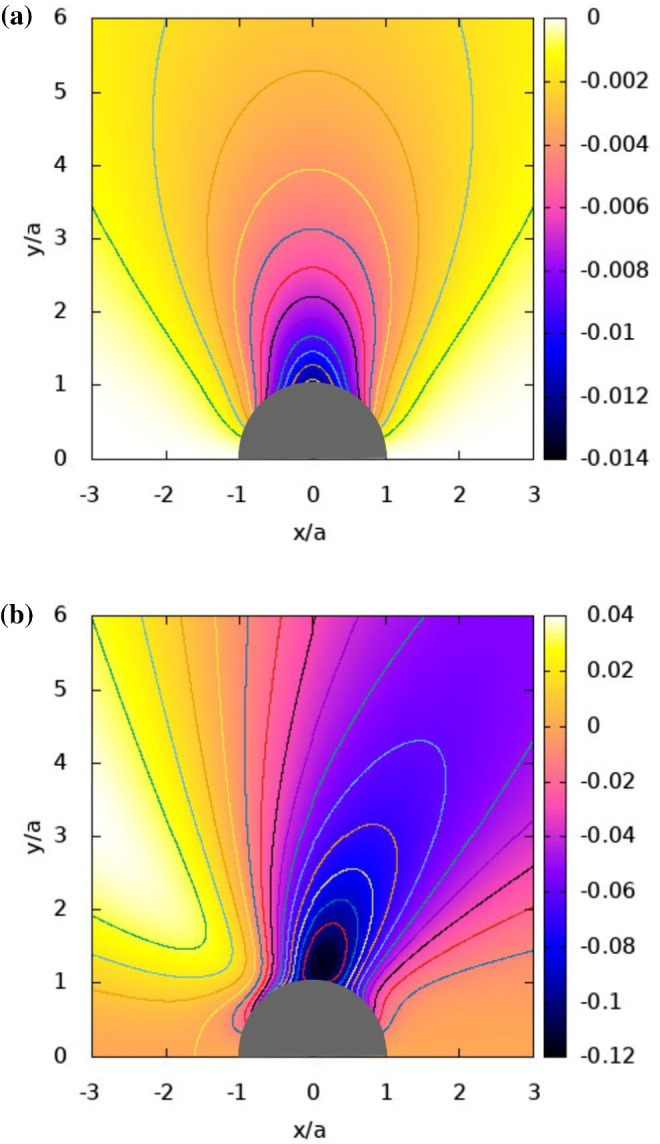
Contours of the stream function normalized by $D_{\mathrm{{T}}}T_{\mathrm{{ref}}}/a^{3}$, and which are plotted for the Janus particles of Fig. 5. ${T}_{\mathrm {ref}}$ is also defined in Fig. 2

**Fig. 7:**
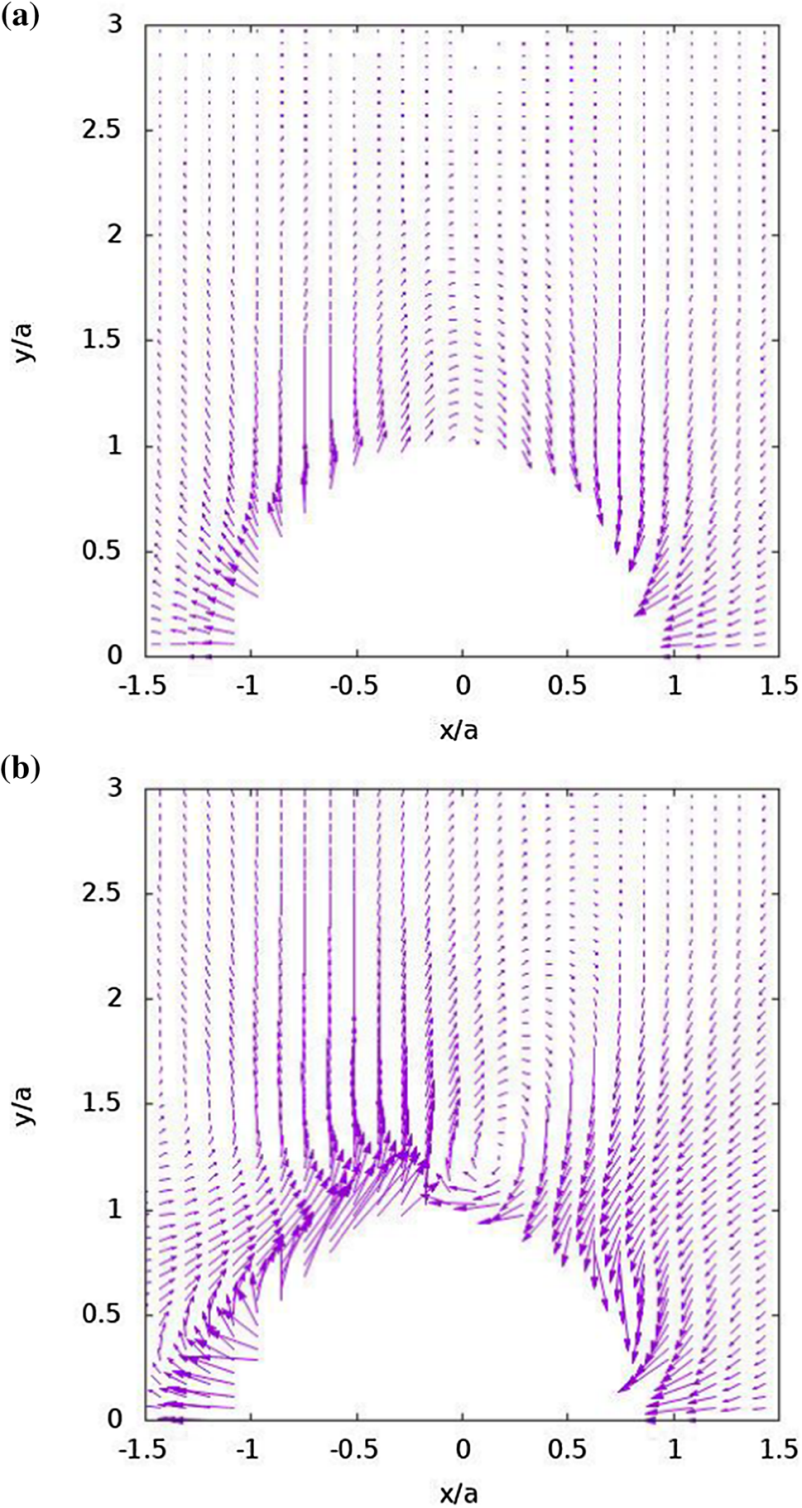
Velocity vectors that are plotted for the cases of Fig. 6, where the vector modulus of each flow field was scaled for optimal presentation and each vector is located at its tail. The velocity field is described in moving coordinates attached to the particle

The JP was taken as embedded in fresh water with ${k}_{\mathrm {O}} = 0.6$ W/(m K). Its southern hemisphere (${x}< 0$) was taken as of Beryllium copper with ${k}_{{2}} = 8$ W/(m K) and the northern hemisphere (${x}> 0$) was taken as polystyrene with ${k}_{{1}} = 0.04$ W/(m K) as in the verification exercise. Since the ratio between the corresponding electric conductivities in this case is rather small (i.e. $\sigma _{1}/\sigma _{2}\sim {10}^{-20})$, ${q}_{{1}}$ was taken as zero while ${q}_{{2}}$ was normalized to one in order to easily normalize the results shown in Figs. 2, 3, 4, 5, 6 and 7. For the sake of comparison, the case of ${k}_{{1}}= {k}_{{2}}=8$ W/(m K), while keeping ${q}_{{1}}=0$, was also investigated. The FL series solution generally converged in less than ten coefficients.

The temperature distributions along the axis of symmetry (*x*) and the normal axis (*y*) are illustrated in Fig. 2. When ${k}_{{1}}= {k}_{{2}} > {k}_{\mathrm {O}}$ (solid line), the northern hemisphere acts as a conductor and thus there is a moderate decrease in the temperature from the heated southern hemisphere of $-1< {x/a}< 0$ to the unheated northern hemisphere of $0< {x/a}< 1$. This finding supports previous approximations of uniform temperature distributions inside the different zones of the JP under similar conditions [11]. However, when ${k}_{{1}}< {k}_{\mathrm {O}} < {k}_{{2}}$ (dashed line), the role of the northern hemisphere as an insulator leads to a smaller surface area, where heat can effectively escape from the southern hemisphere. Thus, a much higher temperature is observed in the southern hemisphere.

The outside temperature ${T}_{\mathrm {O}}$ decays approximately as 1/*r* far away from the JP as illustrated in Fig. 2b and thus far from the particle, it converges towards the homogeneous particle solution of Eq. (A12). The highest temperature along the transverse y direction is found on the axis of symmetry (${y}=0$) as expected. The kinks in the temperature distributions at $| {x/a}| =1$ and ${y/a}=1$ are due to the changes in the thermal conductivity from the particle’s material to the surrounding medium. A similar kink exists at ${x}=0$ in Fig. 2a for ${k}_{{1}}< {k}_{\mathrm {O}} < {k}_{{2}}$ (dashed line) due to the sharp difference between ${k}_{{1}}$ and ${k}_{{2}}$.

The contours of the normalized heat flux modulus are depicted in Fig. 3. The case of ${k}_{{1}}= {k}_{{2}} > {k}_{\mathrm {O}}$, of Fig. 3a, shows that the highest heat flux modulus exists on the boundary of ${x}=0$ inside the JP, where the northern hemisphere acting as a conductor, serves as an escape route for the heat from the heated southern hemisphere. On the other hand, the role of the northern hemisphere as an insulator is demonstrated in Fig. 3b for the case where ${k}_{{1}}< {k}_{\mathrm {O}} < {k}_{\mathrm {2,}}$. As a result, the heat flux modulus significantly increases between the southern hemisphere and the ambient medium just left of ${x}=0$, in order to compensate over the blocking effect of the northern hemisphere. The points of zero heat flux along the axis of symmetry (${y}=0$), correspond to the points of maximum temperature as seen in Fig. 2a.

The distribution of the normalized heat flux along the axis of symmetry is plotted in Fig. 4. For ${k}_{{1}}= {k}_{{2}} > {k}_{\mathrm {O}}$ (solid line) the heat flux is negative for ${x/a}< -0.6$ and positive for ${x/a}> 0.6$, where $x/a\approx -0.6$ is the point of maximum temperature. It shows that heat escapes to the surrounding medium for ${x/a}< -0.6$ while for ${x/a}> -0.6$ the heat escapes towards the unheated northern hemisphere (${x}> 0$). However, for ${k}_{{1}}< {k}_{\mathrm {O}} < {k}_{{2}}$ (dashed line) the blocking effect of the northern hemisphere causes the magnitude of the heat flux at ${x}=-{a}$ to grow, while it is also accompanied with a significant increase in the heat flux towards the surrounding medium near the JP boundary at ${x}=0$ as seen in Fig. 3b.

The heat flux direction pattern is further illustrated in the vector plots of Fig. 5. The magnitude of the heat flux vector field as a whole was adjusted to provide optimal illustration of the flux pattern. The role of the northern hemisphere as an efficient heat conductor in Fig. 5a for ${k}_{{1}}= {k}_{{2}} > {k}_{\mathrm {O}}$ , causes a significant amount of heat to leave the heated southern hemisphere (${x}< 0$) towards the unheated northern hemisphere (${x}> 0$). This is in sharp contrast to the pattern seen in Fig. 5b for ${k}_{{1}}< {k}_{\mathrm {O}} < {k}_{{2}}$, where the insulating effect of the northern hemisphere, causes the heat flux to be redirected towards the y direction and escaping to the surrounding fluid near $(x,y) = (0, a)$. This has a profound effect on the induced velocity field, as discussed later. There is also some readjustment in the heat flux direction in the ambient solute near the JP, as a part of the near and far fields of ${T}_{\mathrm {O}}$. A similar behaviour was already found for homogeneous spheroidal particles [35].

The contours of the normalized Stokes stream function are plotted in Fig. 6. They are depicted for the flow field in a moving frame of reference attached to the JP. As there is no flow inside the particle, the contours are outside the particle. They indicate a circular type of flow from the left of the figure to the right, i.e. from the hot southern pole of the particle to the cold northern pole, which is of similar behaviour found for the induced flow from the hot tip to the cold tip of a stationary spheroidal particle [35]. Strikingly, the case of ${k}_{{1}}= {k}_{{2}} > {k}_{\mathrm {O}}$, shows almost symmetric patterns of the stream-function contours around ${x}=0$ in Fig. 6a, while the case of ${k}_{{1}}< {k}_{\mathrm {O}} < {k}_{{2}}$ shows a slightly tilted pattern in Fig. 6b. The differences between the contour levels of the stream function in Fig. 6b are much higher than in Fig. 6a while still having similar spatial distancing, hence showing much higher velocity magnitudes for the case of ${k}_{{1}}< {k}_{\mathrm {O}} < {k}_{{2}}$ as compared for the case of ${k}_{{1}}= {k}_{{2}} > {k}_{\mathrm {O}}$. This behaviour is also associated with a more profound level of vortical structures as discussed next.

Velocity vector plots are shown in Fig. 7 for the two cases. As in the case of the heat flux plots of Fig. 5, the magnitude of the whole field in each case was adjusted for optimal view, so the vector length should not be compared between Figs. 7a and b, but only between the vectors of the same field. The velocity field for the case ${k}_{{1}}< {k}_{\mathrm {O}} < {k}_{{2 }}$, shown in Fig. 7b, is much more energetic compared to the case ${k}_{{1}}= {k}_{{2}} > {k}_{\mathrm {O}}$ (Fig. 7a), as evidenced by the much more visible vortical structures depicted in Fig. 7b. The overall velocity magnitude level of the case ${k}_{{1}}< {k}_{\mathrm {O}} < {k}_{\mathrm {2,}}$ was found to be 15 times larger than that of ${k}_{{1}}= {k}_{{2}} > {k}_{\mathrm {O}}$. The vortex formation observed above the particle around ${x}=0$ indicates a high level of heat flux as seen in Fig. 5b. It demonstrates how changing the thermal character of the unheated northern hemisphere, from a conductor in the case of ${k}_{{1}}= {k}_{{2}} > {k}_{\mathrm {O}}$ to an insulator in the case of ${k}_{{1}}< {k}_{\mathrm {O}} < {k}_{{2}}$, yields a higher self-propelling velocity $U_{P}$ which is generally accompanied with a more profound vortical structure.

## Comparison of the JP model of two different hemispheres with the metal-coated hemisphere

It is instructive to compare the explicit expression obtained for the thermophoretic mobility of a light-activated spherical JP against some recent experimental measurement of self-induced semi-coated JP. Consider a JP composed of dielectric (polystyrene) and metallic (gold) hemispheres corresponding to indices 1 and 2, respectively, that is immersed in water. The thermal conductivity of gold is $k_{{2}} =318$ W(m K) and that of water is $k_{\mathrm {O}}=0.6$ W/(m K). The thermal conductivity of the polystyrene is much lower than of the gold and so is the electric conductivity. Hence, one can take that $\sigma _{1}/\sigma _{2},~ k_{1}/k_{2},~ k_{1}/k_{O}\ll 1$. Thus following Eqs. (A16), (A17) and (13) one finds

17 $$\begin{aligned} U_{P}\backsimeq \left( 5{aD}_{T}q_{2}k_{2} \right) /\left( 48k_{o} \right) . \end{aligned}$$

The heat power absorbed by a particle can be expressed by the laser power *P* and the conductive volume of the particle. Thus one can write , where  is the particle’s overall volume and  is the volume of the metallic (highly conductive) phase. Hence, $\lambda =1$ for a homogenous particle, $\lambda =1/2$ for the JP of two hemispheres as in Sects. 2–4 and $\lambda =\left( 3t \right) /\left( 2a \right)$ for a semi-coated nanoparticle (NP) where *a* is the radius of the spherical particle and *t* is the thickness of the thin metallic coating. The thermophoretic mobility of polystyrene can be taken as $D_{\mathrm {T,PS}} = 1.82$ $\left( \upmu \mathrm{{m}} \right) ^{2}/\left( \mathrm{{s}}~ \mathrm{{K}} \right)$ and for gold $D_{\mathrm {T,AU}} = 2.88$ $\left( \upmu \mathrm{{m}} \right) ^{2}/\left( \mathrm{{s}}~ \mathrm{{K}} \right)$ [15]. These values are compatible with the value of $D_{\mathrm {T}}$ $\sim 3 \left( \upmu \mathrm{{m}} \right) ^{2}/\left( \mathrm{{s}}~ \mathrm{{K}} \right)$ taken for a dielectric spherical NP [11]. Since the coefficients of the metallic and dielectric phases are of the same order and following ‘Appendix B’, one can approximate for the AU/PS JP $D_{\mathrm {T}} = (D_{\mathrm {T,AU~ }}+D_{\mathrm {T,Ps}})/2 = 2.35$ $\left( \upmu \mathrm{{m}} \right) ^{2}/\left( \mathrm{{s}}~ \mathrm{{K}} \right)$.

Thus using Eq. (17) and  for a spherical particle of radius *a*, one gets;

18 $$\begin{aligned} U_{P}\backsimeq \frac{15}{192\pi }\frac{\lambda D_{T}P}{a^{2}k_{O}}, \end{aligned}$$

which can serve as a practical approximation for estimating the JP thermal mobility. Note that the mobility linearly increases with laser irradiation power *P* and decays with the radius *a* squared in agreement with JP reported measurements of AU/PS [10]. Fig. 2b in Peng et al. [10] displays the mobility of a $5~ \upmu \mathrm{{m}}$ semi-coated AU/PS JP irradiated by light (660 nm) for laser power ranging between 80 and 180 mW. For comparison we choose $P = 140$ mW, $D_{\mathrm {T}} = 2.35$ $10^{{-6}} {\mathrm{{m}}}^{{2}}$/(s K), $k_{\mathrm {O}}=0.6$ W/(m K), $a=2.5~ \upmu \mathrm{{m}}$ and $\lambda =\left( 3t \right) / \left( 2a \right)$. Since the coating thickness *t* is not explicitly given in Ref. [10], we refer to a related work on a semi-coated spherical JP with coating thickness of $t = 2$, 4, 6 nm and same radius [38]. Finally, with $t = 4$ nm and $\lambda =2.4\cdot {10}^{-3}$ we get $U_{P}\simeq 5~ \upmu \mathrm{{m/s}}$ which is in excellent agreement with the experimental value under the same condition given in Fig. 2b [10].

## Conclusions

An analytic model was developed for obtaining the self-thermophoretic mobility of a light-activated Joule-heated JP composing of two distinct material hemispheres (different thermal and electric conductivities) and surface-dependent mobility, embedded in a quiescent conductive liquid. In particular, we investigated the interior temperature distribution within a two-phase laser-heated JP under the electrostatic (Rayleigh) framework, depending on the amount of light irradiation. Also explicitly computed is the thermophoretic mobility of the heated JP as well as the corresponding thermoosmotic Stokes stream function including the detailed velocity and vorticity fields prevailing in the surrounding quiescent conducting fluid. The temperature, velocity and vorticity fields are governed by the linear Poisson equation and the creeping flow (Stokes) model. The induced thermoosmotic flow field is generally driven by a Soret-type slippage with a surface-dependent mobility parameter.

The new JP analytic solution (displaying ‘symmetry breaking’) thus developed is based on using a Fourier–Legendre (FL) series in each one of the corresponding three phases that quickly converged for all the investigated cases. The general analytic procedures outlined in Sect. 3 and ‘Appendices A and B’ are based on using the coefficients of the FL series for the temperature fields in each phase as well as the variable Soret-type slip, followed by solving a linear matrix equation in order to find these unknown coefficients. The particular case of a two-term piecewise-constant Soret slippage is also analysed and it is demonstrated that the common assumption of taking the average between these two values, holds only when the contrast between the JP conductivities is moderate. A somewhat simplified (single-parameter) explicit solution for the thermophoretic mobility of a light-activated JP is found by assuming a constant Soret coefficient. This single- parameter is related to the dipolar term in the FL expansion of the ‘outer’ temperature field. Yet, one can obtain an approximate simple and practical expression for estimating the self-propelling velocity of a heated JP in terms of-its size, laser amplitude (power) and the distinct (constant) conductivities of the various three phases as of Eq. (18). This analytic solution was also compared against the exactly computed and experimental values and was found to be reasonably accurate.

A sharp difference between the two hemispheres’ thermal conductivities led to a sharp temperature decline in the hemisphere acting as an insulator and the creation of noticeable vortical structures around the JP. The present solution may be found useful also in the optimal design of autonomous light-driven micro-swimmers and for enhancing fluid mixing effects by means of opto- (thermal) procedures. One of the next challenges is to incorporate the developed model as a sub-grid model into a multi-scale simulation and analysing the collective behaviour of such interacting micro-photoactive JP motors of arbitrary shapes.

## Appendix A: Fully spectral solution for the temperature solution

We use the FL series for the inner temperature as in Eqs. (7) and (8), but we rewrite the external temperature ${T}_{\mathrm {O}}$ as;

A1 $$\begin{aligned} T_{\mathrm{{O}}}\left( R,\mu \right)= & {} \frac{a^{2}}{6k_{\mathrm{{O}}}R}\left( q_{1}k_{1}+q_{2}k_{2} \right) +\frac{{\overline{C}} _{0}}{R}\nonumber \\&+\sum \limits _{n=1}^\infty {C_{n}R^{-\left( n+1 \right) }P_{n}\left( \mu \right) } . \end{aligned}$$

Equation (A1) is identical to the FL solution in Eq. (11) except the split in the term of *O*(1/*R*). It is shown here that ${\overline{C}}_{0}=0$ and hence ${C}_{\mathrm {O}}$ of Eq. (11) is $C_{\mathrm{{O}}}=a^{2}\left( q_{1}k_{1}+q_{2}k_{2} \right) /\left( 6k_{\mathrm{{O}}} \right)$.

Using a least-square operation, one gets from the boundary conditions of Eqs. (4) and (5);

A2 $$\begin{aligned}&\int _{-1}^0 {T_{2}\left( r=a,\mu \right) P_{n}\left( \mu \right) \mathrm{{d}}\mu } +\int _0^1 {T_{1}\left( r=a,\mu \right) P_{n}\left( \mu \right) \mathrm{{d}}\mu } \nonumber \\&\quad =\int _{-1}^1 {T_{\mathrm{{O}}}\left( r=a,\mu \right) P_{n}\left( \mu \right) \mathrm{{d}}\mu } , \end{aligned}$$

A3 $$\begin{aligned}&k_{2}\int _{-1}^0 {\frac{\partial T_{2}\left( r=a,\mu \right) }{\partial r}P_{n}\left( \mu \right) \mathrm{{d}}\mu } \nonumber \\&\quad +k_{1}\int _0^1 {\frac{\partial T_{1}\left( r=a,\mu \right) }{\partial r}P_{n}\left( \mu \right) \mathrm{{d}}\mu } \nonumber \\&\quad =k_{\mathrm{{O}}}\int _{-1}^1 {\frac{\partial T_{\mathrm{{O}}}\left( r=a,\mu \right) }{\partial r}P_{n}\left( \mu \right) \mathrm{{d}}\mu } . \end{aligned}$$

When substituting the FL series solutions of Eqs. (7), (8) and (A1) into Eqs. (A2) and (A3), one can use the following function;

A4 $$\begin{aligned} \gamma _{m,n}&\equiv \int _0^1 P_{m} \left( \mu \right) P_{n}\left( \mu \right) \mathrm{{d}}\mu \nonumber \\&=\left( -1 \right) ^{m+n}\int _{-1}^0 P_{m} \left( \mu \right) P_{n}\left( \mu \right) \mathrm{{d}}\mu , \end{aligned}$$

where $\gamma _{m,n}=0$ if $\left( m\ne n \right)$ and both *m* and *n* are either odd or even numbers [38–40], otherwise;

A5 $$\begin{aligned} \gamma _{2m+1,2n}=&\frac{\left( -1 \right) ^{m+n+1}}{4^{m+n}}\nonumber \\&\times \frac{\left( 2n \right) !\left( 2m \right) !}{\left( n! \right) ^{2}\left( m! \right) ^{2}\left( 2n+2m+2 \right) \left( 2n-2m-1 \right) }.\nonumber \\ \end{aligned}$$

Equation (A2), which is the result of requiring continuity of temperature on the particle’s surface yields;

A6 $$\begin{aligned} {\overline{C}}_{0}=0,A_{0}=\frac{1}{2}\left( \frac{k_{2}}{k_{1}}-1 \right) \sum \limits _{n=0}^\infty A_{2n+1} \gamma _{2n+1,0}, \end{aligned}$$

and for ${m}> 0$ we get;

A7 $$\begin{aligned} C_{m}= & {} A_{m}+F_{m}+\left( m+\frac{1}{2} \right) \left( -1 \right) ^{m}\left( 1-\frac{k_{2}}{k_{1}} \right) \nonumber \\&\sum \limits _{n=0} {A_{2n+1}\gamma _{2n+1,m}} , \end{aligned}$$

where

A8 $$\begin{aligned} F_{m}= & {} \frac{a^{2}\left( q_{1}-q_{2} \right) }{6}\left[ \left( -1 \right) ^{m}\left( m+\frac{1}{2} \right) \left( \gamma _{m,0}+2\gamma _{m,2} \right) \right. \nonumber \\&\left. -\frac{1}{2}\delta \left( m \right) -\delta \left( m-2 \right) \right] , \end{aligned}$$

and $\delta \left( m \right)$ is the Dirac delta function.

Similarly, by using Eq. (A5) which is the result of requiring continuity of heat flux normal to the JP’s surface, one gets;

A9 $$\begin{aligned} -C_{m}= & {} \frac{k_{2}}{k_{\mathrm{{O}}}}\frac{m}{m+1}A_{m}+G_{m}\nonumber \\&+\frac{2m+1}{m+1}\frac{k_{2}}{k_{\mathrm{{O}}}}\left( 1-\frac{k_{1}}{k_{1}} \right) \left( -1 \right) ^{m}\sum \limits _{n=0}^\infty {{nA}_{2n}\gamma _{2n,m}} ,\nonumber \\ \end{aligned}$$

where

A10 $$\begin{aligned} G_{m}= & {} \frac{a^{2}}{6k_{\mathrm{{O}}}}\left( q_{1}k_{1}-q_{2}k_{2} \right) \left[ \left( -1 \right) ^{m}\gamma _{m,0}\frac{2m+1}{m+1}-\delta \left( m \right) \right] \nonumber \\&+\frac{a^{2}}{6k_{\mathrm{{O}}}}\left( q_{1}-q_{2} \right) \left[ \left( -1 \right) ^{m}\frac{2m+1}{m+1}\left( k_{1}+k_{2} \right) \gamma _{m,2}\right. \nonumber \\&\left. -\frac{2}{3}k_{1}q_{1}\delta \left( m-2 \right) \right] . \end{aligned}$$

Next, combining Eqs. (A7) and (A9) yields the following matrix equation for the *A*’s:

A11 $$\begin{aligned}&-\left( 1+\frac{k_{2}}{k_{\mathrm{{O}}}}\frac{m}{m+1} \right) A_{m}=F_{m}+G_{m}\nonumber \\&\quad + \left( -1 \right) ^{m}\frac{2m+1}{m+1}\left( 1-\frac{k_{1}}{k_{2}} \right) \nonumber \\&\quad \left[ \frac{m+1}{2}\sum \limits _{n=0}^\infty {A_{2n+1}\gamma _{2n+1,m}} +\frac{k_{2}}{k_{1}}\sum \limits _{n=0}^\infty {{nA}_{2n}\gamma _{2n,m}} \right] .\nonumber \\ \end{aligned}$$

It is also worth noting from Eqs. (A8) and (A11) that for $m=0$ one gets $\gamma _{0,0}=1$ and thus ${F}_{{0}}= {G}_{{0}}=0$. Hence, Eq. (A6) directly follows from Eqs. (A7) and (A9).

One interesting outcome of Eq. (A11) is that for the particular case of $k_{{1}}=k_{{2}}=k$, we get a rather simple explicit solution for the *A*’s and *C*’s as follows:

A12 $$\begin{aligned} A_{m}=-\left( 1+\frac{k}{k_{\mathrm{{O}}}}\frac{m}{m+1} \right) ^{-1}\left( F_{m}+G_{m} \right) , \end{aligned}$$

and according to Eq. (A7) one also finds for ${m}> 0$;

A13 $$\begin{aligned} C_{m}=A_{m}+F_{m}. \end{aligned}$$

Next, assuming in addition that ${q}_{{1}}= {q}_{{2}}={q}$, implies that ${F}_{{m}}= {G}_{{m}}=0$ and also ${A}_{{m}}= {C}_{{m}}=0$. Thus, one recovers the known temperature solution for a single homogeneous spherical particle [22];

A14 $$\begin{aligned}&T_{1}\left( r \right) =T_{2}\left( r \right) =-\frac{q}{6}\left( r^{2}-a^{2}-\frac{2ka^{2}}{k_{\mathrm{{O}}}} \right) , \end{aligned}$$

A15 $$\begin{aligned}&T_{\mathrm{{O}}}\left( r \right) =\frac{qa^{3}k}{3k_{\mathrm{{O}}}r}. \end{aligned}$$

Finally, by truncating the infinite series, one obtains a relatively simple and practical approximation for ${C}_{{1}}$ which governs the JP self-induced thermophoretic mobility $U_{\mathrm{{P}}}$ by Eq. (13). The coefficient ${C}_{{1}}$ displays the ‘symmetry-breaking’ effect. It depends on the contrast between the thermal ($k_{1}\ne k_{2})$ and electric ($\sigma _{1}\ne \sigma _{2})$ conductivities of the two-face JP, as well as the amplitude ${\vert }E_{0}{\vert }$ of light irradiation, where ,  is the volume of the spherical particle and ${i}=1,2$.

Thus, one finally finds from Eqs. (A7) and (A9) that;

A16 $$\begin{aligned} C_{1}\simeq \frac{F_{1}k_{1}/k_{\mathrm{{O}}}-G_{1}\left( 1+k_{1}/k_{2} \right) }{1+k_{1}/k_{o}+k_{1}/k_{2}}, \end{aligned}$$

where following Eqs. (A8), (A10) and using $\gamma _{1,0}=1/2$, $\gamma _{1,2}=1/8$ ;

A17 $$\begin{aligned}&F_{1}=\frac{3a^{2}}{16}\left( q_{1}-q_{2} \right) =\frac{3a^{2}}{16}{\vert E_{0}\vert }^{2}\frac{\sigma _{1}}{k_{1}}\left( 1-\frac{\sigma _{2}k_{1}}{\sigma _{1}k_{2}} \right) ,\nonumber \\ \end{aligned}$$

A18 $$\begin{aligned}&G_{1}=\frac{a^{2}}{8k_{\mathrm{{O}}}}\left[ q_{2}k_{2}-q_{1}k_{1}+\frac{1}{4}\left( q_{2}-q_{1} \right) \left( k_{1}+k_{2} \right) \right] \nonumber \\&\quad \,\,=a^{2}\frac{\vert E_{0}\vert ^{2}}{8k_{\mathrm{{O}}}}\left[ \sigma _{2}-\sigma _{1}+\frac{1}{4}\left( \frac{\sigma _{2}}{k_{2}}-\frac{\sigma _{1}}{k_{1}} \right) \left( k_{1}+k_{2} \right) \right] . \nonumber \\ \end{aligned}$$

Equation (A16) can serve as a simple and quick estimate for the amplitude of the self-thermophoretic JP mobility in terms of the relevant physical parameters and also determines conditions for velocity reversal (i.e. change of sign). As an example, for the parameters used in Figs. 2b and 7b; (${k}_{{1}}$, ${k}_{{2}}$, ${k}_{\mathrm {O}}) =$ (0.04, 8, 6) W/(m K) and ${q}_{{1}}=0$, Eq. (A16) renders $C_{1}/T_{\mathrm{{ref}}}=-0.26280$, compared against the exact numerical value of $C_{1}/T_{\mathrm{{ref}}}=-0.29565$ where $T_{\mathrm{{ref}}}=q_{2}a^{2}k_{2}/\left( 3k_{o}\right)$.

## Appendix B: Thermophoresis due to non-uniform slip

For the sake of completeness, we also provide a new explicit solution for the self-thermophoresis problem of a light-activated JP due to a non-uniform Soret-type slip velocity. Towards this goal, we assume that the mobility coefficient $D_{\mathrm{{T}}}$ in Eq. (6) varies along the surface of the JP such that;

B1 $$\begin{aligned} D_{\mathrm{{T}}}\left( \theta \right) =\sum \limits _{m=1}^\infty \lambda _{m} \frac{{\mathrm{{d}}P}_{m}\left( \mu \right) }{\mathrm{{d}}\mu }, \end{aligned}$$

where $\lambda _{m}$ are prescribed coefficients. For a constant slip, i.e. constant $D_{\mathrm{{T}}}$, one has $\lambda _{m}=D_{\mathrm{{T}}}\delta \left( m-1 \right)$. Substituting Eqs. (B1) and (12) in Eq. (6) leads to;

B2 $$\begin{aligned} V_{S}\left( \theta \right) =\frac{\mathrm{{sin}}\theta }{a}\sum \limits _{n=1}^\infty \sum \limits _{m=1}^\infty \lambda _{m} C_{n}\frac{{\mathrm{{d}}P}_{m}\left( \mu \right) }{\mathrm{{d}}\mu }\frac{{\mathrm{{d}}P}_{n}\left( \mu \right) }{\mathrm{{d}}\mu },\nonumber \\ \end{aligned}$$

where $V_S$ is the modulus of the velocity $v_S$ defined in Eq. (6). Taking advantage of Bailey’s [41] expression for the product of two associated Legendre functions, Eq. (B2) can also be written as;

B3 $$\begin{aligned} V_{S}\left( \theta \right)= & {} \frac{\mathrm{{sin}}\theta }{a}\sum \limits _{n=1}^\infty \sum \limits _{m=1}^\infty \sum \limits _{s=o}^{min\left( m-1,n-1 \right) } \alpha _{s} \left( m,n \right) \nonumber \\&\lambda _{m}C_{n}\frac{{\mathrm{{d}}P}_{m+n-1-2s}\left( \mu \right) }{\mathrm{{d}}\mu }, \end{aligned}$$

where

B4 $$\begin{aligned}&\alpha _{s}\left( m,n \right) \nonumber \\&\quad =\frac{2}{\pi }\frac{\varGamma \left( s+3 / 2 \right) \varGamma \left( m-s+1/2 \right) \varGamma \left( n-s+1 / 2 \right) \left( m+n-s \right) !\left( m+n-2s-2 \right) !\left( 2m+2n-4s-1 \right) }{s!\Gamma \left( m+n-s+1/2 \right) \left( m-s-1 \right) !\left( n-s-1 \right) !\left( m+n-2s \right) !}, \end{aligned}$$

and $\varGamma \left( n \right)$ denotes the Gamma function.

Following the hydrodynamic reciprocal theorem [42], the JP mobility can be readily found by averaging the Soret slip velocity of Eq. (B3) over the spherical surface resulting in;

B5 $$\begin{aligned} U_{\mathrm{{P}}}= & {} -\frac{2}{3a}\sum \limits _{n=1}^\infty \sum \limits _{m=1}^\infty \sum \limits _{s=o}^{min\left( m-1,n-1 \right) } \alpha _{s} \left( m,n \right) \nonumber \\&\lambda _{m}C_{n}\delta \left( m+n-2s-2 \right) , \end{aligned}$$

due to orthogonality of the associated Legendre functions. Note that only combinations of where m$+$n and $|m - n|$ is even, contribute to the triple sum in Eq. (B5). A more compact form of Eq. (B5) can be obtained by substituting Eq. (B4) and executing the delta operation leading to;

B6 $$\begin{aligned} U_{\mathrm{{P}}}=-\frac{2}{3a}\sum \limits _{n=1}^\infty \sum \limits _{m=1}^\infty \beta \left( m,n \right) \lambda _{m}C_{n}, \end{aligned}$$

where

B7 $$\begin{aligned}&\beta \left( m,n \right) =\beta \left( n,m \right) \nonumber \\&\quad =\alpha _{s}\left( m,n \right) \delta \left( m+n-2s-2 \right) \nonumber \\&\quad =\frac{3}{\pi }\frac{\left( \frac{m+n}{2} \right) \left( \frac{m+n}{2}+1 \right) \varGamma \left( \frac{m-n}{2}+\frac{3}{2} \right) \varGamma \left( \frac{n-m}{2}+\frac{3}{2} \right) }{\left( \frac{m-n}{2} \right) !\left( \frac{n-m}{2} \right) !\left( m+n+1 \right) }.\nonumber \\ \end{aligned}$$

One can easily verify that $\beta \left( 1,1 \right) =1$ and letting $\lambda _{m}=D_{\mathrm{{T}}}\delta \left( m-1 \right)$ in Eq. (B6) reduces to the constant slip value given in Eq. (13).

By following the same procedure for evaluating the Stokes stream function of Eq. (14), one finds that by virtue of Eq. (B3) the generalized stream function under a non-uniform slip can accordingly be written in a body frame of reference as;

B8 $$\begin{aligned}&\psi \left( r,\theta \right) =-\frac{1}{2}a^{2}{sin}^{2}\theta \left[ U_{\mathrm{{P}}}\left( \frac{a}{r} \right) +S \right] , \end{aligned}$$

B9 $$\begin{aligned}&S=\frac{1}{a}\mathop \sum \limits _{n=1}^\infty \mathop \sum \limits _{m=1}^\infty \mathop \sum \limits _{s=o}^{\min \left( m-1,n-1 \right) } \alpha _{s} \left( m,n \right) \nonumber \\&\quad \lambda _{m}C_{n}\left( \frac{a}{r} \right) ^{m+n-1-2s}\frac{{\mathrm{{d}}P}_{m+n-1-2s}\left( \mu \right) }{\mathrm{{d}}\mu }\left[ \left( \frac{r}{a} \right) ^{2}-1 \right] \nonumber \\&\quad H\left( m+n-3-2s \right) , \end{aligned}$$

where H(n) denotes the Heaviside’s unit function (${H}({n})=0$ for ${n}< 0$ and ${H}({n})=1$ for $n\geqslant 0)$ and the thermophoretic mobility ${U}_{\mathrm {p}}$ is given in Eq. (B6). It is worth mentioning that for the limiting case of a constant Soret coefficient, i.e. $\lambda _{m}=D_{\mathrm{{T}}}\delta \left( m-1 \right)$, by letting ${m}=1$ and ${s}=0$ in Eqs. (B8) and (B9) we recover Eq. (14).

Before concluding this ‘Appendix’, let us examine the case of a piecewise continuous slip on a JP, often discussed in the literature, e.g. [15], where for a spherical JP, it is assumed that;

B10 $$\begin{aligned} D_{\mathrm{{T}}}\left( \theta \right) =\sum \limits _{m=1}^\infty \lambda _{m} \frac{{\mathrm{{d}}P}_{m}\left( \mu \right) }{\mathrm{{d}}\mu }=\left\{ {\begin{array}{*{20}c} D_{\mathrm{{T}}}^{\left( 1 \right) }~ , &{} 0\leqslant \mu \leqslant 1\\ D_{\mathrm{{T}}}^{\left( 2 \right) }~ , &{} -1\leqslant \mu <0\\ \end{array} } \right\} ,\nonumber \\ \end{aligned}$$

where in general the two constants $D_{T}^{\left( 1 \right) }\ne D_{T}^{\left( 2 \right) }$. Multiplying Eq. (B10) with $\left( 1-\mu ^{2} \right) {\mathrm{{d}}P}_{n}\left( \mu \right) /\mathrm{{d}}\mu$ and integrating over the surface of the two JP hemispheres leads to;

B11 $$\begin{aligned}&\sum \limits _{m=1}^\infty \lambda _{m} \int _{-1}^1 {\left( 1-\mu ^{2} \right) \frac{{\mathrm{{d}}P}_{m}\left( \mu \right) }{\mathrm{{d}}\mu }\frac{{\mathrm{{d}}P}_{n}\left( \mu \right) }{\mathrm{{d}}\mu }\mathrm{{d}}\mu } \nonumber \\&\quad =D_{\mathrm{{T}}}^{\left( 1 \right) }\int _0^1 {\left( 1-\mu ^{2} \right) \frac{{\mathrm{{d}}P}_{n}\left( \mu \right) }{\mathrm{{d}}\mu }\mathrm{{d}}\mu } \nonumber \\&\qquad + D_{\mathrm{{T}}}^{\left( 2 \right) }\int _{-1}^0 {\left( 1-\mu ^{2} \right) \frac{{\mathrm{{d}}P}_{n}\left( \mu \right) }{\mathrm{{d}}\mu }\mathrm{{d}}\mu } . \end{aligned}$$

After integration by parts, we get;

B12 $$\begin{aligned} \lambda _{m}= & {} D_{\mathrm{{T}}}^{\left( 1 \right) }\delta (m-1)+ \frac{2m+1}{2} \left( D_{\mathrm{{T}}}^{\left( 1 \right) }-D_{\mathrm{{T}}}^{\left( 2 \right) } \right) \gamma _{m1},\nonumber \\ \end{aligned}$$

where $\gamma _{n,m}$ are the auxiliary functions defined in Eqs. (A4) and (A5). Clearly for a constant mobility, i.e. $D_{\mathrm{{T}}}^{\left( 1 \right) }=D_{\mathrm{{T}}}^{\left( 2 \right) }=D_{\mathrm{{T}}}$ one gets as expected $\lambda _{m}=D_{\mathrm{{T}}}\delta \left( m-1 \right)$. Hence taking a constant ${D}_{\mathrm {T}}$ means that only the first term on the right hand side of Eq. (B12) is accounted. Finally, it is important to note that the presented formulations in this ‘Appendix’ are accurate for any surface axisymmetric-dependent Soret-type coefficient.
